# Early asymptomatic graft failure in coronary artery bypass grafting: a study based on computed tomography angiography analysis

**DOI:** 10.1186/s13019-023-02199-0

**Published:** 2023-04-05

**Authors:** Zengqiang Han, Guodong Zhang, Yu Chen

**Affiliations:** 1 Cardiac Surgery Department, Peking University People’s Hospital, Beijing, 100044 China; 2 Thoracic Surgery Department, Shandong Cancer Hospital Affiliated to Shandong First Medical University, Jinan, China

**Keywords:** Coronary artery bypass grafting, Graft failure, Asymptomatic, Computed tomography angiography

## Abstract

**Background:**

Asymptomatic graft failure after coronary bypass grafting surgery (CABG) may have negative impact on the patients’ short- and long-term outcomes. Cardiac computed tomography angiography (CTA) has been proved to be another choice to detect graft failure besides coronary artery angiography in several studies. We aimed to identify the rate and predictors of asymptomatic graft failure detected by CTA before discharge.

**Methods and results:**

A total of 955 grafts of 346 consecutive asymptomatic patients who received CTA examination after CABGs were included in this retrospective study from July 2017 to Dec 2019. We divided 955 grafts into the patent group and occluded group by CTA results. Logistic regression model at graft-level were established to determine predictors of the early asymptomatic graft occlusion. The overall asymptomatic graft failure rate was 4.71% (45/955), and there was no difference between the arterial and venous conduits in different target territories (*P* > 0.05). The logistic regression at graft-level analysis showed that female (OR 3.181, CI 1.58–6.40, *P* = 0.001), composite grafting (OR 6.762, CI 2.26–20.28, *P* = 0.001), pulse index value (OR 1.180, CI 1.08–1.29, *P* < 0.001) and new postoperative atrial fibrillation (POAF) (OR2.348, CI 1.15–4.78, *P* = 0.018) were independent risk factors that affect graft failure, while early postoperative dual-antiplatelet treatment with aspirin and clopidogrel was a protective factor (OR 0.403, CI 0.19–0.84, *P* = 0.015).

**Conclusions:**

Early asymptomatic graft failure is associated with both patient and surgical factors including female gender, high PI value, composite graft strategy and the new POAF. However, the early dual- antiplatelet therapy with aspirin and clopidogrel may be useful for preventing graft failure.

**Supplementary Information:**

The online version contains supplementary material available at 10.1186/s13019-023-02199-0.

## Introduction

Coronary artery bypass grafting (CABG) surgery is a widely used revascularization strategy for complex multivessel coronary artery disease (CAD) that provides symptomatic relief and increases the long-term survival in patients with CAD [1, 2]. Owing to some patients do not have symptoms or clinical signs of myocardial ischemia [3], few studies about early asymptomatic graft failure have been reported and graft failure rates remain unclear [4]. However, early asymptomatic graft failure may have negative impact on the patients’ short- and long-term outcomes and develop symptoms when exercise increase, because the relevant myocardial area are still unsupplied [5, 6]. Computed tomography angiography (CTA) scanners combine a high spatial resolution with the ability to demonstrate the anatomy through volume-rendered images, thus producing a more sensitive evaluation than does conventional or spiral CT. Cardiac CTA, as a low-invasive investigation method for the evaluation of the early grafts has been proved to be another choice besides coronary artery angiography in several studies [7–9]. CTA examination in our center was routinely used in patients who underwent CABG prior to discharge. This study aimed to identify the rate and predictors of early silent graft failure detected by CTA before discharge.

## Patients and methods

### Patients selection

**Fig. 1:**
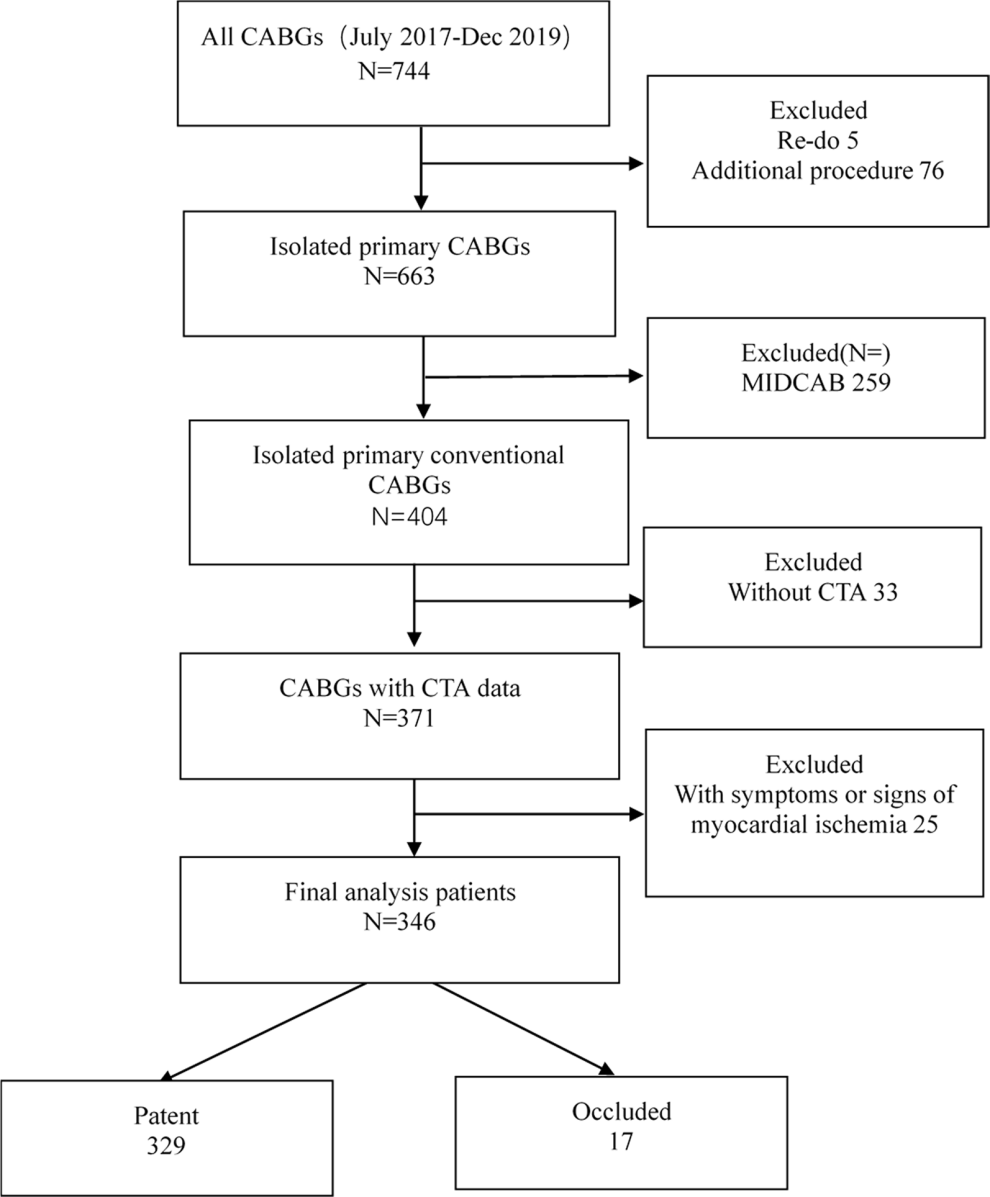
Data flowchart for patients included in the study. CABG, Coronary artery bypass grafting; MIDCAB, Minimally Invasive Direct Coronary Artery Bypass Grafting; CTA, computed tomography angiography

Data for isolated CABG were retrospectively collected from July 1, 2017, to Dec 31, 2019, from the Peking University People’s Hospital database. There were 744 patients who underwent CABGs; we excluded 5 who underwent redo surgeries, 76 who underwent concomitant additional procedures, 259 who underwent minimally invasive direct coronary artery bypass grafting (MIDCAB) for single-vessel disease, 25 who had symptoms or clinical signs of myocardial ischemia and 33 without CTA data due to renal dysfunction. The total 346 patients were divided into two groups: the patent group was defined as all anastomoses were patent and the occluded group was defined as at least one occluded anastomoses (Fig. 1). This study was approved by our institutional Review Board /Ethics Committee. Consent for individual use of data was waived because of the nature of the study and previous approval for the use of such data at the time of operative consent.

### Surgical methods

All patients underwent CABGs through a median sternotomy. Stabilization of the target coronary arteries was accomplished with a tissue stabilizer (Octopus, Medtronic Corporation, Minneapolis, MN) and an intra-coronary shunt (Medtronic Corporation, Minneapolis, MN) was used during off-pump CABGs. In on-pump CABGs, cardiopulmonary bypass was established after standard ascending aorta cannulation and 2-stage venous cannulation of the right atrium, all distal anastomoses were performed after cardiac arrest. All procedures were performed by surgeons who had expertise in both on-pump and off-pump CABG, defined as more than 5 years of experience after residency training and completion of more than 100 cases of the specific type of surgery. The parameters were measured by The VeriQ system transit time flow measurement (TTFM) device (MediStim Inc, Oslo, Norway). The parameter yielded by TTFM system included the mean graft flow volume (MGF), the PI (pulse index) and the diastolic flow fraction (DF). Satisfactory blood flow parameters criteria: The shape of blood flow waveform is stable and repeatable; PI < 5; MGF > 15 ml/min. If sufficient graft flow was not obtained, graft revision was considered and performed until diastolic graft flow was confirmed. All patients were given heparin 2500U per 4 h postoperatively, and then received antiplatelet therapy with aspirin, clopidogrel or both when tracheal intubation removed.

After the drainage tube was removed, patients without contraindications received cardiac CTA prior to discharge. They were evaluated by Dual Source CT scanner SOMATOM Force CCTA (Siemens Healthineers, Erlangen, Germany). CCTA images were analysed with GE AW 4.6 advance Workstation (GE Healthcare, Chicago, IL, USA) and were evaluated by 2 independent experienced radiologists. The number of grafts was counted by distal anastomoses, and the lesions of grafts and anastomoses were evaluated according to the FitzGibbon classification system. An occlusion of a graft was diagnosed if the graft was not filled by contrast medium along its whole length.

### Related definitions

Early asymptomatic graft failure: Graft failure in patients who do not have symptoms or clinical signs of myocardial ischemia (elevated myocardial enzyme met the perioperative myocardial infarction diagnostic criteria, electrocardiogram change) before discharge. Composite grafts: Y-SVGs: Y composite graft with a saphenous veins (SVG); I, Y-IMAs and SVG: an arteriovenous I or Y conduit with internal mammary artery (IMAs) and SVG; Y-IMAs: Y composite graft with LIMA and RIMA.

### Statistical analysis

Continuous variables were expressed as median and interquartile range (IQR) because most of the data were not normally distributed. Nominal and categorical variables were given as absolute numbers and proportions (%). The Mann–Whitney U-test was used for analysis of numerical data. Binary data obtained with Pearson’s χ2 test or Fischer’s exact test as appropriate. Intra-group analysis, the Bonferroni correction was adopted when appropriate.. Preoperative baseline data and perioperative data were included in the univariate analysis. Measurements of different grafts within the same patient were considered statistically independent. The variables with a univariable *P* value < 0.05 or near 0.05 and variables based on clinical judgment were tested in a multivariable model using Logistic regression and ORs to determine the independent predictors of the occurrence of graft occlusion at graft-level. Optimal cutoff values of MGF, PI and DF to predict early graft failure, were determined by means of the ROC curve analysis. *P* < 0.05 was considered statistically significant. All the analyses were performed with SPSS version 26.0 and RStudio.

## Results

Among a total of 346 patients (955 grafts) included in our study, 17 (45 grafts) had graft occlusion in the CT scans at 4–34 days before discharge after CABG. Among the 45 occluded grafts, there were 11 IMAs and 34 SVG grafts. Among which, there were two cases with four, 9 cases with three, 4 cases with 2 and 2 cases with 1 occluded grafts. There was no significant difference in patency rates among the different target territories (*P* = 0.266). We also compared the patency rates of the arterial and venous conduits in different target territories, and found that there was no difference between the arterial and venous conduits in different target territories (*P* > 0.05) (Additional file 1: Table S1).

Baseline characteristics of the patent group and the occluded group at graft-level are given in Table 1. The occluded group had almost twice the amount of female (48.9% vs 27.5%, *P* = 0.003), and had a higher level in triglycerides (1.6 vs 1.5 mmol/L, *P* = 0.024).Baseline procedural characteristics and postoperative data of the patent group and the occluded group at graft-level are demonstrated in Table 2. The postoperative TNI max (3.0 vs 1.8 ng/ml, *P* = 0.022) and the postoperative CK-MB max (16.5 vs 12.8 U/L, *P* = 0.032) of the occluded group were both significantly higher than the patent group. The occluded group has higher PI (3.4 vs 2.4, *P* < 0.001), but lower DF (63% vs 69%, *P* = 0.007). There was significant difference between the groups in antiplatelet treatment strategy (*P* = 0.02). There was no significant difference between the two groups in other baseline procedure data (*P* > 0.05).

**Table 1:** Baseline Patient Characteristics of the Patent group and the Occluded group at graft-level

	Patent (910)	Occluded (45)	*P*
Age	64 (57–69)	65 (58–69)	0.819
Female (n, %)	250 (27.5)	22 (48.9)	0.003
BMI	25.3 (23.1–27.4)	25.0 (23.1–27.7)	0.924
Hypertension (n, %)	601 (66.0)	30 (66.7)	1.000
Diabetes (n, %)	359 (39.5)	20 (44.4)	0.536
Insulin treatment	104 (11.4)	9 (20)	0.095
Hyperlipidemia (n, %)	459 (50.4)	25 (55.6)	0.544
Previous stroke (n, %)	147 (16.15)	4 (8.89)	0.217
Smoking (n, %)	245 (26.92)	12 (26.67)	1.000
PVD (n, %)	128 (14.07)	9 (20.00)	0.275
Ventricular aneurysm (n, %)	28 (3.08)	2 (4.44)	0.648
Previous MI (n, %)	159 (17.47)	7 (15.56)	0.843
PCI	136 (14.95)	6 (13.33)	0.835
Arrhythmia (n, %)	20 (2.20)	0 (0.00)	0.619
AMI (n, %)	243 (15.71)	12 (26.67)	1.000
Diseased vessels	3.0 (3.0–3.0)	3.0 (3.0–3.0)	0.251
Left main disease (n, %)	202 (22.20)	7 (15.56)	0.358
NYHA (n, %)			0.399
I	3 (0.33)	0 (0.00)	
II	699 (76.81)	36 (80.00)	
III	201 (22.09)	8 (17.78)	
IV	7 (0.77)	1 (2.22)	
LVEF (%, ± s)	63.6 (55.6–68.7)	65.3 (58.2–69.8)	0.354
LVEF < 40% (n, %)	29 (3.19)	2 (4.44)	0.654
EuroSCORE II	1.0 (0.7–1.5)	1.1 (0.7–1.4)	0.744
Triglycerides	1.5 (1.1–2.1)	1.6 (1.4–2.5)	0.024
PLT	199.0 (166.0–245.0)	185.0 (154.0–229.5)	0.111
Preoperative creatinine (μmol/ml, ± s)	73.0 (62.0–87.0)	69.0 (58.0–79.5)	0.059

*BMI* body mass index, *COPD* chronic obstructive pulmonary disease, *PVD* peripheral vascular diseases, *PCI* percutaneous coronary intervention, *AMI* acute myocardial infarction, *NYHA* New York Heart Association, *LVEF* left ventricular ejection fraction, *LVEDd* left ventricular end-diastolic dimension, *PLT* platelet count

**Table 2:** Procedural characteristics of the Patent group and the Occluded group at graft-level

Items	Patent (910)	Occluded (45)	*P*
On-pump	406 (44.62)	23 (51.11)	0.444
SVG harvest technique			0.072
Endoscopic	103 (11.32)	11 (24.44)	
Open	443 (48.68)	23 (51.11)	
Proximal anastomosis technique			0.079
PAC	151 (16.59)	7 (15.56)	
Proximal anastomosis device	282 (30.99)	20 (44.44)	
SAC	115 (12.64)	8 (17.78)	
No-touch	362 (39.78)	10 (22.22)	
Grafting strategy			
In situ IMAs	354 (38.90)	9 (20.00)	0.000
AO-SVG	530 (58.24)	30 (66.67)	
Composited	19 (2.09)	6 (13.33)	
Free IMAs	7 (0.77)	0 (0.00)	
IABP			0.383
No-use	820 (90.11)	38 (84.44)	
Pre-operation	42 (4.62)	4 (8.89)	
Intra-operation	30 (3.30)	2 (4.44)	
Post-operation	13 (1.43)	0 (0.00)	
Number of vessel conduits	3.0 (3.0–4.0)	4.0 (3.0–4.0)	0.198
PI	2.4 (1.8–3.2)	3.4 (2.3–5.1)	0.000
MGF	26.0 (18.0–39.0)	23.0 (14.0–39.0)	0.152
DF	69.0 (63.0–76.0)	63.0 (51.8–74.3)	0.007
Bleeding	500 (350–800)	500 (300–900)	0.812
Postoperative TNI max	1.8 (0.8–4.4)	3.0 (1.4–4.4)	0.022
Postoperative CK-MB max	12.8 (6.2–23.2)	16.5 (9.7–28.9)	0.032
Mechanical ventilation time	11.0 (8.0–18.0)	11.5 (8.0–17.0)	0.525
Operation time	270.0 (240.0–310.0)	265.0 (240.0–307.5)	0.914
Antiplatelet strategy			0.022
Aspirin	427 (46.92)	30 (66.67)	
Clopidogrel	33 (3.63)	2 (4.44)	
Dual-antiplatelet	443 (48.68)	12 (26.67)	
New POAF (n, %)	232 (25.49)	17 (37.78)	0.077
CRRT (n, %)	13 (1.43)	1 (2.22)	0.487

*LIMA* left internal mammary artery, *SVG* saphenous vein grafting, *AO* aorta, *PI* pulse index, *MGF* mean graft flow, *DF* diastolic flow fraction, *IABP* intra-aortic balloon pump, *POAF* postoperative atrial fibrillation, *CRRT* continuous renal replacement therapy, *PAC* partial aorta clamp, *SAC* single aorta clamp

**Fig. 2:**
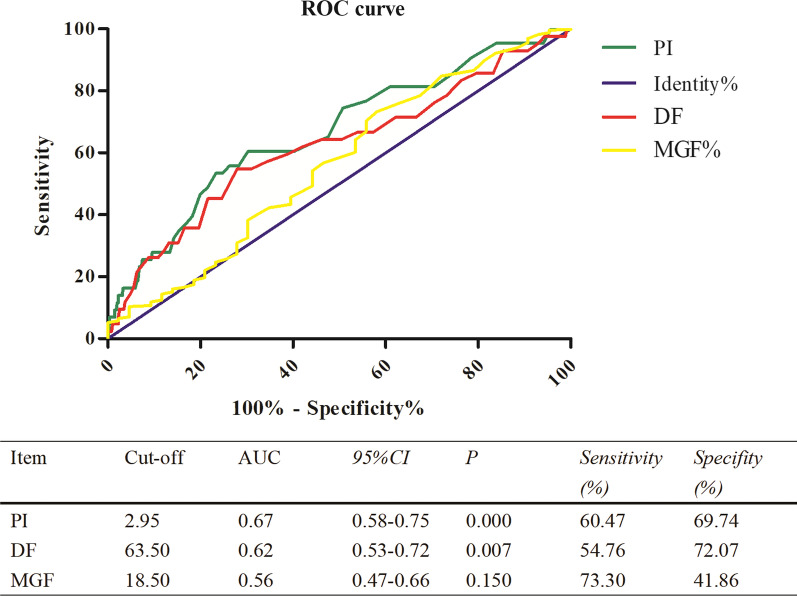
The ROC analysis representing the cut-off TTFM values for predicting early graft failure. TTFM, transit time flow measurement; PI, pulse index; MGF, mean graft flow; DF, diastolic flow fraction; CI: confidence limit

The results of ROC analysis of TTFM parameters are presented in Fig. 2. The cut-off values for detecting occluded grafts were a PI value > 2.95 (*P* < 0.001) and DF < 63.5% (*P* = 0.007), however MGF < 18.5 ml/min was not found to be a statistically significant indicator of graft failure (*P* = 0.056).

The intra-group comparison of grafting strategy demonstrate that the patency rate of in situ IMAs group(97.5% vs 76.0%, Bonferroni *P* < 0.0083) and AO-SVG group(94.6% vs 76.0%, Bonferroni *P* < 0.0083) were both significant higher than the composite grafts group (Additional file 1: Table S2).

**Fig. 3:**
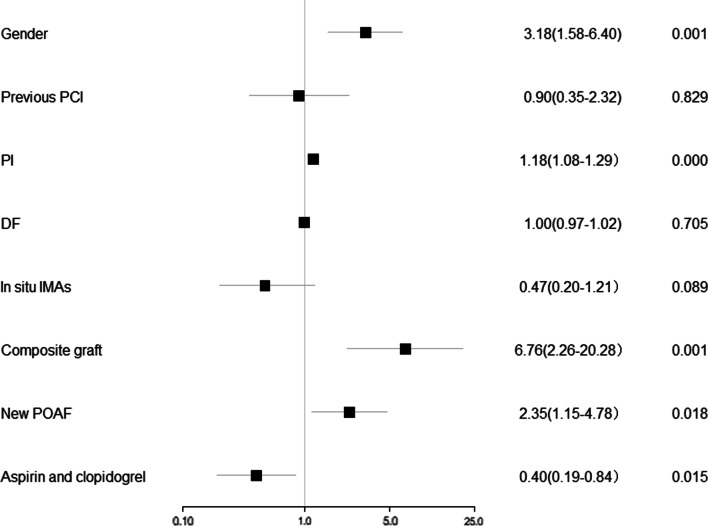
Logistic regression results at graft-level. PCI, percutaneous coronary intervention; PI, pulse index; DF, diastolic flow fraction; IMA, internal mammal artery

Then we built a graft-level logistic regression model for multiple-factor regression analysis and results demonstrated that female gender (OR 3.181, CI 1.58–6.40, *P* = 0.001), composite grafting (OR 6.762, CI 2.26–20.28, *P* = 0.001),PI (OR 1.180, CI 1.08–1.29, *P* < 0.001) and new POAF (OR 2.348, CI 1.15–4.78, *P* = 0.018) were independent risk factors that affect graft failure, while early postoperative dual-antiplatelet treatment with aspirin and clopidogrel was a protective factor (OR 0.403, CI 0.19–0.84, *P* = 0.015) (Fig. 3).

## Discussion

Interest in understanding the factors associated with graft failure after CABG has been longstanding, but little study about the immediate (before discharge), asymptomatic graft failure factors and its occlusion rates. Previous studies have reported 1-year vein graft failure rates of 10–20%, limited data suggest that a substantial proportion of such occlusions occur much earlier [10, 11]. One major finding of our study was that in-hospital graft occlusion occurred in 4.91% of patients and 4.71% of grafts, compares favorably with the rate of 11% reported by Alicja Zientara and the rate of 7% published by Nakano [4, 12]. The reason for the difference may be that our study has included the arterial graft. And we found that there was no difference of the graft failure among different target territories (*P* > 0.05) and there was no difference between the arterial and venous conduits (*P* = 0.059).

In this study, graft failure occurred predominantly in female gender, which was similar to that reported in previous literature [4]. Transit time flow measurement is a common method for intraoperative assessment of the adequacy of a bypass graft, and it was added to the European guidelines for revascularization in 2014 [13]. In this study, the higher PI value (OR 1.180, *P* = 0.000) was confirmed to be a predictive parameter of graft failure, which was similar to the results (OR 1.3, *P* = 0.31) reported by Gabriele Di Giammarco et al. [14]. Optimal cutoff values of PI determined by means of the ROC curve analysis was 2.95 in this study. The cut-off value of 5 for an optimal graft is suggested by the guidelines, while some surgeons have consider a PI under 3 as an indicator of a good graft, which is accordance with our study [13, 14]. The value of DF was a risk factor for graft failure in the univariate analysis, but not a risk factor in the multivariate regression variate analysis. The composite grafting strategy was confirmed to be significant risk factor for early silent graft failure. In intra-group analysis, the patency rates of in situ IMAs subgroup (97.5% vs 76.0%, *P* = 0.000) and the AO-SVG subgroup (94.6% vs 76.0%, *P* = 0.003) were both significantly higher than the composite grafts subgroup. In our study, there were total 25 composite grafts, of which 6 grafts was occluded including 3 Y-SVGs, 2 I, Y-LIMA and SVG, and 1 Y-IMAs. Although arterial composite grafts have been demonstrated to be both safe and effective for revascularization, studies assessing the safety and efficacy of using saphenous veins as a composite graft have produced conflicting results [15, 16]. One such study recommended against the use of a saphenous vein composite graft given that it could steal flow from the stem graft and lead to suboptimal short-term stem patency outcomes, especially the LIMA [17]. In this study, composite grafts including arterial composite grafts and saphenous vein composite graft, were confirmed to be a risk factor of short-term patency. However, the sample of this study was small, further larger sample study are needed to confer our results.

An interesting finding of our study was that the dual-antiplatelet treatment with aspirin and clopidogrel was a protective factor of graft failure. The optimal antiplatelet strategy following CABG remains controversial, aspirin is considered the preferred antiplatelet drug to prevent graft failure after CABG. However, there is emerging evidence on the potential benefits of dual antiplatelet therapy with aspirin and clopidogrel or ticagrelor after CABG, including to prevent graft occlusion and adverse cardiac events post CABG [18–20]. Graft failure results from complex pathophysiological processes that can lead to complete occlusion of the graft. Progression of the pathophysiological alterations of graft failure involves several distinct phases. Primary graft failure is characterized by thrombosis in the early phase (within hours to 1 month after grafting), addition of a P2Y12 inhibitor is thought to help preserve graft patency and reduce adverse cardiac events by inhibiting platelet mediated progression of graft disease [21–23]. A surprising finding of our study was that new POAF might have negative impact on the early graft patency. POAF is common following cardiac surgery, which is regarded as benign, transient and self-limited, it has been associated with increased morbidity, thromboembolic events and an increased duration and cost of hospitalization [24]. However, few studies have reported the POAF have some negative impact on the graft patency, further study about the relationship between POAF and graft failure are needed.

Although we used the TTFM to confirm the quality of anastomoses, and if sufficient graft flow was not obtained, graft revision was considered and performed until diastolic graft flow was confirmed. There were still 17 of 346 patient have at least one occluded graft. However only 7 patient received percutaneous coronary angiography examination to verify the CTA-detected occlusion and were successfully re-intervened, considering the myocardial area of them remain unsupplied and might cause adverse cardiac event because of early graft failure.

### Limitations

Several limitations of our study should be recognized. The first and most important limitation of this study was its descriptive nature, using a relatively small cohort of patients at a single institution. Second, the degree of the native coronary stenosis and collateralization of chronically occluded coronaries might affect the graft patency were not included in our study.

## Conclusions

Early asymptomatic graft failure is associated with both patient and surgical factors including female gender, high PI value, composite graft strategy and the new POAF. However, the early postoperative dual-antiplatelet therapy with aspirin and clopidogrel may be useful for preventing graft failure. Postoperative CTA scan can help to identify the early asymptomatic graft failure and to make further reinterventions.

## Supplementary Information

**Additional file 1.** Tables about the patency rates and grafting strategy.

## Data Availability

Data will be made available on request.
