# Supplementary material for: Early asymptomatic graft failure in coronary artery bypass grafting: a study based on computed tomography angiography analysis
Source: J Cardiothorac Surg. 2023 Apr 5;18:98. doi: 10.1186/s13019-023-02199-0 (PMC10074891; doi:10.1186/s13019-023-02199-0)
Supplement: Supplementary file 1 — Additional file 1. Tables about the patency rates and grafting strategy. [file 13019_2023_2199_MOESM1_ESM.docx]

**Additional file 1**

Table Supplement 1. The patency rates of different target territories

| Item | Conduit | | Patency | | *P* | *P* |
| --- | --- | --- | --- | --- | --- | --- |
| LAD (358) | |  | | 346(96.64) |  |  |
|  | | IMAs (328) | | 319(97.26) |  |  |
|  | | SVG (30) | | 27(90.00) | 0.070 |  |
| DIAGs (107) | |  | | (96.26) |  |  |
|  | | IMAs (27) | | 27(100.00) |  |  |
|  | | SVG (80) | | 76(95.00) | 0.355 |  |
| PDA/PL/RCA (301) | |  | | 285(94.68) |  |  |
|  | | IMAs (14) | | 12(85.71) |  |  |
|  | | SVG (287) | | 273(95.12) | 0.166 |  |
| LCX/OM (188) | |  | | 175(93.09) |  |  |
|  | | IMAs (17) | | 17(100.00) |  |  |
|  | | SVG (172) | | 159(92.44) | 0.612 | 0.266 |

LAD, left anterior descending artery; DIAG: diagonal branch; PDA, posterior descending branches; PL, posterior descending branches; RCA: right coronary artery; OM: obtuse marginal; LCX: left circumﬂex artery;

Table Supplement 2. Intra-group analysis about grafting strategy

| Item | Numbers | | Patency | | *P* |
| --- | --- | --- | --- | --- | --- |
| In situ IMAs | | 354/365 | | 97.5 |  |
| AO-SVG | | 530/560 | | 94.6 | 0.034 |
| In situ IMAs | | 9/365 | | 97.5 |  |
| AO-IMAs | | 7/7 | | 100 | 1.000 |
| In situ IMAs | | 9/365 | | 97.5 |  |
| Composite graft | | 19/25 | | 76.0 | 0.000 |
| AO-SVG | | 529/559 | | 94.6 |  |
| Composite graft | | 6/25 | | 76.0 | 0.003 |
| AO-IMAs | | 7/7 | | 100 |  |
| AO-SVG | | 529/559 | | 94.6 | 1.000 |
| AO-IMAs | | 7/7 | | 100 |  |
| Composite graft | | 6/25 | | 76.0 | 0.296 |

IMA, internal mammary artery; AO, aorta; SVG, saphenous vein grafting
